# Surgical consent during the COVID19 pandemic: Saving lives while in crisis editorial

**DOI:** 10.1016/j.amsu.2020.07.039

**Published:** 2020-07-26

**Authors:** Evander Meneses, Mark McKenney, Dessy Boneva, Adel Elkbuli

**Affiliations:** Department of Surgery, Division of Trauma and Surgical Critical Care, Kendall Regional Medical Center, Miami, FL, USA; Department of Surgery, Division of Trauma and Surgical Critical Care, Kendall Regional Medical Center, Miami, FL, USA; University of South Florida, Tampa, FL, USA; Department of Surgery, Division of Trauma and Surgical Critical Care, Kendall Regional Medical Center, Miami, FL, USA; University of South Florida, Tampa, FL, USA; Department of Surgery, Division of Trauma and Surgical Critical Care, Kendall Regional Medical Center, Miami, FL, USA

**Keywords:** Medical ethics, Surgical consent, Emergency surgery, Trauma surgery, COVID-19 pandemic

## Abstract

• Compares obtaining informed consent from a non-COVID-19 patient versus a COVID-19 person under investigation or confirmed positive in order to maintain healthcare workers safety and minimize PPE use.
• Explains the use of technology in the form of video chat to aid in informed consent from healthcare surrogates of patients who are unable to provide their own informed consent.
• Discusses alternative solutions to obtaining informed consent from a COVID-19 person under investigation or confirmed positive.

Autonomy, justice, beneficence, and non-maleficence are the four basic principles of medical ethics, these pillars guide healthcare workers in delivering treatment to their patients. Informed consent falls under the principle of autonomy in which the caregiver provides the information and evidence of a particular course of treatment to the patient in order for the patient to determine their care. For informed consent to be valid, three components are required: disclosure, capacity, and voluntariness [1,2]. Disclosure pertains to the evidence given to the patient that they are able to understand. Capacity is the ability of the patient to comprehend and form a reasonable course of events based on the potential consequences of their decision. Voluntariness refers to the patient's right to make their own decision without any undo external influences.

With the COVID-10 pandemic caused by the virus named, severe acute respiratory syndrome coronavirus 2 (SARS-CoV-2) there has been a significant strain in healthcare resources, requiring restructuring of hospital systems in order to optimally utilize care for those afflicted by the deadly virus. With the strain in resources, surgical cases have taken a sharp decline in number. Additionally, the pandemic has posed a unique barrier to informed consent to the trauma patient population.

Our hospital is a 417-bed Level 1 American College of Surgeons (ACS) verified Trauma Center providing care to the residents of Miami-Dade County. As a pediatric and adult trauma center, our patient population spans all ages and injuries range from a single isolated injury requiring no surgical intervention to a severe polytrauma patient who requires multiple trips to the operating room with a multidisciplinary team of surgeons to provide life-saving treatment. Our hospital also performs a wide array of non-urgent, elective surgeries, most of which have been halted to allocate the appropriate resources to urgent and emergent surgeries. Informed consent has been a unique challenge during this pandemic for the trauma patient as the risks of undergoing surgery versus foregoing surgery until a later date can significantly affect the hospital course and road to recovery.

The full pathophysiology caused by SARS-CoV-2 can be quite variable. Most commonly, symptomatic patients present with respiratory symptoms, however gastrointestinal and hematologic symptoms have also been observed [3,4]. A combination of any of these symptoms also occurs. How this affects the potential surgeries that trauma patients may undergo is not completely known and so this is one of the aspects that we inform our patients during the consenting process.

## 1 Obtaining informed consent

**Fig. 1:**
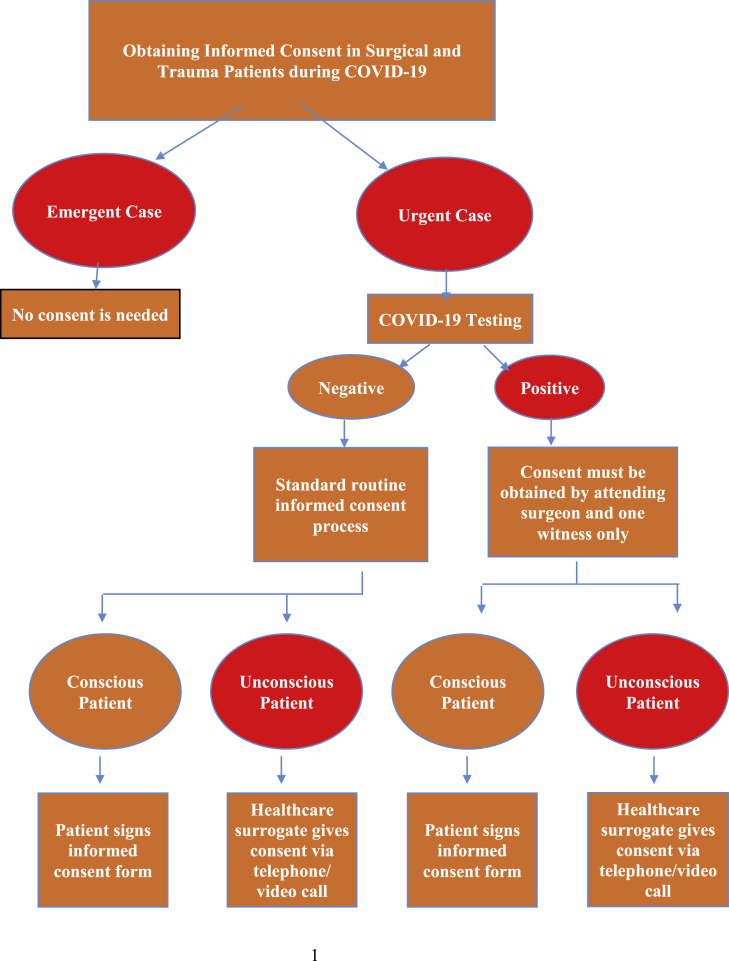
Obtaining informed surgical consent during the COVID19 pandemic.

At our institution, all patients who are admitted are tested for COVID-19 and results return usually within 24 hours. For emergent surgeries on trauma patients near death from hemorrhage, we proceed to the operating room without consent, as is the case at any trauma center. In this situation the patient is in extremis and lacks capacity due to exsanguination and altered mental status. For urgent surgeries, however we must obtain informed consent. We follow the ACS principles for informed consent, providing the patient information pertaining to the nature of their illness and consequence of no treatment, nature of proposed operation, commonly known complications, alternative forms of treatment, and a discussion of the different types of qualified medical providers who will participate in the care [5]. At our institution, the surgical resident and attending surgeon are the initial contact to the patient and/or family members when obtaining informed consent for surgical procedures. The attending surgeon provides information to the patient and/or family members and signs the physical consent form prior to the patient rolling into the operating suite. We now additionally speak with the patient and/or family members regarding COVID-19, explaining that we are unsure how it may impact their perioperative morbidity and possible mortality. We also explain that there is a risk for transmission within the hospital as well as further transmission after hospital discharge if they were to become infected in the hospital (Fig. 1).

## 2 COVID-19 negative

Trauma and surgical patients who have tested negative for COVID-19 after a confirmatory SARS-CoV-2 test undergo the same usual informed consent process established at our institution prior to the pandemic and described above. The biggest challenge has been obtaining consent from the surrogate (e.g., next of kin) whenever the patient is unable to give their own consent due to severe injury. Our hospital implemented the “no visitors” policy early during the pandemic, where visitors are not allowed to visit patients to minimize COVID-19 transmission risk to staff and patients’ families. Due to this policy, surgical consent for patients unable to consent must be obtained from the surrogate over the phone. While obtaining consent via telephone was not uncommon before the pandemic, as often times the surrogate would not be able to stay at the hospital at all times, the gravity of the situation must be carefully explained, as now the surrogate has not seen the patient since the traumatic event. By using video chat, we have been able to allow the surrogate to see the patient and made fully aware of the serious nature of their condition in order to determine care for the patient after discussing about the treatment options.

## 3 COVID-19 person under investigation or confirmed positive

Our hospital has dedicated areas where COVID-19 patients are being treated whether it is a specific section of the intensive care units or a specific section of the medical/surgical floors. We have had to change the way we consent patients who are COVID-19; person under investigation (PUI) or confirmed positive. Trauma and surgical patients who are a PUI or confirmed positive case undergo the informed consent process only by the attending trauma surgeon who will be operating on the patient. The surgical resident no longer helps obtain consent from the patient for the trauma attending. There are several reasons why we have established this process. This minimizes the amount of traffic entering these COVID-19 positive areas. In addition, this limits the number of healthcare workers exposed to these patients as well as reduces the number of PPE used. Similarly, when requiring consent from a surrogate, the use of video chat is utilized in order to convey the severity of injury and the seriousness of the situation in order to provide the healthcare surrogate with the appropriate information to give consent for the patient. These changes that we have made have been easily implemented as now it is only the trauma attending obtaining the informed consent, instead of the surgery resident followed by the trauma attending. While we are a training program and we understand that it is important for residents to learn to discuss risks and benefits of a proposed operation, we are temporarily suspending this COVID-19 related practice in order to fully utilize our resources to take care of all hospitalized patients. In the near future when the number of COVID-19 patients starts to stabilize and decline, residents can then go back to consenting the COVID-19 patients or PUIs for procedures using the appropriate PPE that will no longer be in short supply. Essentially, we are able to prevent overwhelming of our hospital's resources by taking these important steps. Ultimately, we are able to ensure the safety of our staff and patients as well as allocate PPE resources effectively and appropriately, especially when there is a scarcity of PPE and the risk of contracting COVID-19 may force healthcare providers out of work to quarantine. In a time where healthcare providers are themselves at risk of becoming a scarce resource, it is important to not only protect and care for the patients but also to provide those same standards to healthcare providers [6].

COVID-19 has posed a unique challenge to the informed consent process of trauma patients. The main problem is the possibility of contracting the virus while hospitalized for a traumatic injury, and further transmission after hospital discharge. While we are still learning about COVID-19, the trauma team must convey to the trauma patient that there is still an unknown amount of risk that comes with COVID-19 if the patient were to fall ill from this disease but that their traumatic injury needs to be addressed even in times of a pandemic.

## 4 Conclusion

In unprecedented times like the present in which healthcare workers dedicate their lives to taking care of the sick despite the risk of falling very ill themselves, it is important to have best practices available. When patients are suspected to have or confirmed to have COVID-19, we limit the exposure and amount of PPE use for that patient when consenting for surgery by having only the attending obtain consent. In patients who are unable to provide their own consent, we use video chat in order to obtain consent from the next of kin in order to convey the severity of the illness and the need for operation.

## Provenance and peer review

Not commissioned, externally peer reviewed.

## Funding

None.

## Declaration of competing interest

Authors declare no competing interests.
